# In Vitro Studies to Define the Cell-Surface and Intracellular Targets of Polyarginine-Conjugated Sodium Borocaptate as a Potential Delivery Agent for Boron Neutron Capture Therapy

**DOI:** 10.3390/cells9102149

**Published:** 2020-09-23

**Authors:** Atsushi Fujimura, Seiji Yasui, Kazuyo Igawa, Ai Ueda, Kaori Watanabe, Tadashi Hanafusa, Yasuaki Ichikawa, Sachiko Yoshihashi, Kazuki Tsuchida, Atsunori Kamiya, Shuichi Furuya

**Affiliations:** 1 Department of Physiology, Okayama University Graduate School of Medicine, Dentistry and Pharmaceutical Sciences, 2-5-1 Shikata-cho, Kita-ku, Okayama 700-8558, Japan; kamiyaa@okayama-u.ac.jp; 2 Neutron Therapy Research Center, Okayama University, 2-5-1 Shikata-cho, Kita-ku, Okayama 700-8558, Japan; pmq06frs@s.okayama-u.ac.jp (S.Y.); igawakazuyo@okayama-u.ac.jp (K.I.); wooai@okayama-u.ac.jp (A.U.); kaori_watanabe@okayama-u.ac.jp (K.W.); hanafusa@okayama-u.ac.jp (T.H.); yiyi08@gmail.com (Y.I.); p1xv8fht@okayama-u.ac.jp (S.F.); 3 Graduate School of Engineering, Nagoya University, Furo-cho, Chikusa-ku, Nagoya 464-8603, Japan; s-yoshihashi@energy.nagoya-u.ac.jp (S.Y.); tsuchida@phi.phys.nagoya-u.ac.jp (K.T.)

**Keywords:** boron neutron capture therapy (BNCT), BSH-polyR, CD44, translational machinery, bioinformatics

## Abstract

Boron neutron capture therapy (BNCT) requires pharmaceutical innovations and molecular-based evidence of effectiveness to become a standard cancer therapeutic in the future. Recently, in Japan, 4-borono-L-phenylalanine (BPA) was approved as a boron agent for BNCT against head and neck (H&N) cancers. H&N cancer appears to be a suitable target for BPA-BNCT, because the expression levels of L-type amino acid transporter 1 (LAT1), one of the amino acid transporters responsible for BPA uptake, are elevated in most cases of H&N cancer. However, in other types of cancer including malignant brain tumors, LAT1 is not always highly expressed. To expand the possibility of BNCT for these cases, we previously developed poly-arginine peptide (polyR)-conjugated mercaptoundecahydrododecaborate (BSH). PolyR confers the cell membrane permeability and tumor selectivity of BSH. However, the molecular determinants for the properties are not fully understood. In this present study, we have identified the cluster of differentiation 44 (CD44) protein and translational machinery proteins as a major cell surface target and intracellular targets of BSH-polyR, respectively. CD44, also known as a stem cell-associated maker in various types of cancer, is required for the cellular uptake of polyR-conjugated molecules. We showed that BSH-polyR was predominantly delivered to a CD44^High^ cell population of cancer cells. Once delivered, BSH-polyR interacted with the translational machinery components, including the initiation factors, termination factors, and poly(A)-biding protein (PABP). As a proof of principle, we performed BSH-polyR-based BNCT against glioma stem-like cells and revealed that BSH-polyR successfully induced BNCT-dependent cell death specifically in CD44^High^ cells. Bioinformatics analysis indicated that BSH-polyR would be suitable for certain types of malignant tumors. Our results shed light on the biochemical properties of BSH-polyR, which may further contribute to the therapeutic optimization of BSH-BNCT in the future.

## 1. Introduction

Boron neutron capture therapy (BNCT) is a cancer therapeutic modality that utilizes a combination of boron ^10^B delivery and epithermal neutron irradiation. It results in a spatially and temporally controlled intracellular ^10^B nuclear reaction, which generates high-energy alpha particles [1,2,3]. To achieve a safe and efficient therapeutic response, high specificity and selectivity of the ^10^B distribution to cancer cells is required.

Current BNCT clinical research primarily utilizes two kinds of boron agents, 4-borono-L-phenylalanine (BPA) and mercaptoundecahydrododecaborate (BSH), whose pharmacokinetics are different. BPA is a ^10^B-derivative of tyrosine/phenylalanine (C_9_H_12_BNO_4_) and is taken up via the L-type amino acid transporter 2 (LAT2) and LAT1 [4,5]. Although these transporters are ubiquitously expressed in the human body, BPA-BNCT has been used to treat several types of cancer, such as head and neck (H&N) cancer and malignant brain tumors [6]. Indeed, BPA was approved by the Ministry of Health, Labour and Welfare of Japan as a boron delivery agent for BNCT of advanced or recurrent H&N cancers in Japan on March 25, 2020.

BSH as well as its derivative carboranes are other candidate boron delivery agents for BNCT. BSH has a high content of boron atoms per single molecule ((B_12_H_11_SH)Na_2_), but it has a critical disadvantage for clinical usage, namely, poor cellular uptake. In the history of boron agent development, BSH preceded BPA, as BSH was applied to the clinical research of BNCT against malignant brain tumors in the 1960–1970s [7], while BPA has been used for melanoma since the 1980s [8]. However, BSH was soon replaced by BPA, because the clinical outcomes in brain tumor patients were not as good, due to its poor cellular uptake. In 2009, Kawabata et al. reported that a modified BNCT protocol in combination with BPA and BSH with X-ray irradiation significantly improved the median survival time (MST) of malignant brain tumor patients [9]. This result indicates that BSH could pass through vulnerable tumor vessels from the blood into tumor tissue (in other words, by an enhanced permeability and retention (EPR) effect).

To expand the possibility of BSH usage in BNCT, numerous studies developing cell-permeable compounds of BSH or carborane were performed. For example, Kirihata and his colleagues recently developed a Kojic acid (KA)-BSH and showed that KA-BSH-BNCT successfully improved survival in a glioma xenograft rat model [10]. Matsumura and his colleagues developed a α-D-mannopyranoside-conjugated closo-dodecarborate compound and demonstrated longer tumor retention compared to BSH and BPA [11]. Hosmane et al. developed carborane-appended 5-thio-D-glucopyranose derivatives [12] and *Nido*-carboranyl levodopa [13], which further expanded the possibility of carboranes.

We previously developed poly arginine (polyR)-conjugated BSH, which could successfully deliver BSH into cancer cells both in vitro and in vivo [14]. Generally, cell-penetrating peptides (CPPs), such as polyR and human immunodeficiency virus (HIV)-derived trans-activator of transcription protein (TAT) peptides consist of serial amino acid sequences of lysine (K) or arginine (R), whose charge is positive [15]. CPPs allow proteins, nucleotides, and small molecules to penetrate the plasma membrane of various kinds of cells in a macropinocytosis-dependent mechanism [16].

Although the molecular determinants of the cell-penetrating properties of CPPs are not fully understood, previous studies identified several cell-surface targets that were required for the membrane-permeability of certain types of CPP. For example, Tanaka et al. showed that arginine 12-mer peptide (R12), but not 8-mer bound to C-X-C chemokine receptor type 4 (CXCR4), further stimulated the macropinocytosis of the R12-conjugated molecule [17]. The intracellular targets for both CPP and BSH are currently not well defined. In the BNCT research field, such information would be helpful to develop new boron delivery agents, since we might be able to predict the efficiency of boron delivery and the efficacy of BNCT by combining this with the information of protein expression profiles of target cancer cells. The purpose of this report is to present experimental evidence supporting this concept.

## 2. Materials and Methods

### 2.1. Cell Culture and Lentivirus Preparation

Human glioma cell lines U87MG and U251MG, human breast cancer cell lines MCF7 and MDA-MB-231, human pancreatic cancer cell lines CFPAC1 and PANC1, and murine mammary gland cell line NMuMG, were cultured in Dulbecco’s modified Eagle’s medium (DMEM) (FUJIFILM Wako Pure Chemical Corporation, Osaka, Japan) with 10% fetal bovine serum (FBS) (Corning Inc., Corning, NY, USA) and penicillin/streptomycin/L-glutamine (Fujifilm-Wako, Japan) as previously described [18]. U87MG, MDA-MB-231, CFPAC-1, PANC-1, and NMuMG were obtained from the American Type Culture Collection (ATCC, Manassas, VA, USA) (catalogue number: HTB-14, HTB-26, CRL-1918, CRL-1469, and CRL-1636, respectively). U251MG and MCF7 were obtained from the Japanese Collection of Research Bioresources Cell Bank (JCRB, Ibaraki, Japan) (catalogue number: IFO50288 and JCRB0134, respectively).

The human glioblastoma stem-like cell lines MGG4, MGG8, MGG18, and MGG23 were established and kindly provided by Dr. Hiroaki Wakimoto (Massachusetts General Hospital). Wakimoto and his colleagues investigated the molecular properties of these cells and characterized MGG4 as “endothelial proliferation”, MGG8 as “primitive neuroectodermal tumor (PNET)-like component”, MGG18 as “giant cell glioblastoma multiforme (GBM)”, and MGG23 as “gemistocytic GBM” [19]. These cells were cultured in neurobasal medium (Thermo Fisher Scientific, Inc., Waltham, MA, USA) with 1 × B-27 supplement (Thermo Fisher Scientific, Inc., Waltham, MA, USA), 1 × N-2 supplement (Thermo Fisher Scientific, Inc., Waltham, MA, USA), and penicillin/streptomycin, or DMEM/F-12 medium (FUJIFILM Wako Pure Chemical Corporation, Osaka, Japan) supplemented with bovine serum albumin, insulin, and transferrin (BIT) 9500 (STEMCELL Technologies, Vancouver, Canada) and penicillin/streptomycin/L-glutamine.

For knockdown experiments, we transfected pLKO.1-puro-shRNA vector (10 µg), psPAX2 (7.5 µg), and pMD2.G (2.5 µg) with TransIT-LT1 (Takara Bio Inc., Kusatsu, Japan) into human embryonic kidney 293FT cells cultured in a 10 cm dish. All parental plasmids were obtained from Addgene (Watertown, MA, USA) (pLKO.1 puro; #8453, psPAX2; #12260, pMD2.G; #12259). After 16 h of transfection, the medium was switched to DMEM with 10% FBS and cultured for 48 h. Then, virus-containing culture supernatant was filtered with a 0.45 µm pore size polysulfone membrane (S-2504) (KURABO INDUSTRIES LTD., Osaka, Japan). For the infection of cancer cells cultured on a 60 mm dish, we added 1 mL of virus-containing culture supernatant and 4 mL of DMEM with 10% FBS. After 2 days of infection, the cells were re-seeded for analysis.

For transformation of the NMuMG cells, we prepared virus particles of pTomo-HRas/shRNA-p53 (Vector plasmid was a kind gift from Dr. Dinorah Morvinski, Tel Aviv University), pTomo (Addgene, #26291), FUW-tetO-wtYAP (Addgene, #84009), and FUdeltaGW-rtTA (Addgene, #19780), and infected cells in the same manner as written above. After the infection of pTomo viruses (pTomo-HRas/shRNA-p53 was used for malignant transformation, with pTomo for a negative control), the cells were further treated with adenovirus harboring Cre recombinase (cat#ADV-005) (Cell Biolabs Inc., San Diego, CA, USA), to activate the HRas expression cassette. The transformation was confirmed by the acquisition of the spheroid-forming capacity and morphological change (epithelial to spindle form).

### 2.2. Sequence of shRNA and Oligonucleotides

The sequences of the shRNA targeting human cluster of differentiation 44 (CD44)#1: 5′-GGACCAATTACCATAACTATTCTCGAGAATAGTTATGGTAATTGGTCC-3′, human CD44#2: 5′-CCTCCCAGTATGACACATATTCTCGAGAATATGTGTCATACTGGGAGG-3′, and negative control: 5′-CCTAAGGTTAAGTCGCCCTCGCTCGAGCGAGGGCGACTTAACCTTAGG-3′ (Addgene, #1864).

### 2.3. Boron Compounds and Boron Content Measurement

**Figure 1:**
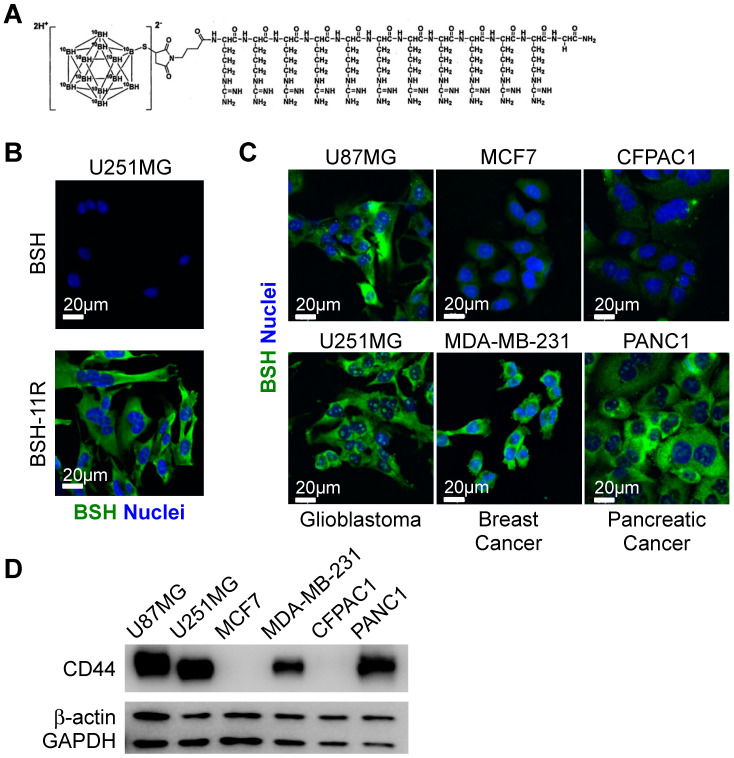
The enhanced cellular uptake of poly-arginine peptide mercaptoundecahydrododecaborate (BSH-polyR) correlates with the expression profile of cluster of differentiation 44 (CD44). (**A**) Scheme of BSH-11R. (**B**) BSH-11R, but not BSH, can penetrate the plasma membrane of U251MG cells. (**C**) BSH-11R efficiently penetrates the cellular membrane of U87MG, U251MG, MDA-MB-231, and PANC1 cells, but not MCF7 and CFPAC1. (**D**) The expression profile of CD44 is correlated with the efficacy of BSH-11R.

BSH and BSH-polyR were kindly provided to us by Dr. Hideki Matsui (Okayama University). BSH was purchased from Katchem (Prague, Czech Republic), and BSH-polyR was synthesized at GlyTech, Inc. (Kyoto, Japan) with thiol-maleimide coupling between BSH and the peptide (Figure 1A) as previously reported [20]. To measure the ^10^B concentration in cells, the cells were washed with phosphate-buffered saline (PBS) twice, followed by dissolution in HNO_3_. After filtration (F2513-16) (Thermo Fisher Scientific, Inc., Waltham, MA, USA), the ^10^B concentration was measured by inductively coupled plasma-atomic emission spectrometry (ICP-AES, Vista Pro) (Seiko Instruments Inc., Chiba, Japan) as previously reported [21] or inductively coupled plasma-mass spectrometry (ICP-MS, 7500cx) (Agilent Technologies Inc., Santa Clara, CA, USA).

### 2.4. Immunomagnetic Positive Selection of BSH-11R-Binding Cells

The immunomagnetic separation of BHS-polyR-binding cells was performed using an EasySep “Do-It-Yourself” positive selection kit (STEMCELL Technologies, Vancouver, BC, Canada) according to the manufacturer’s instruction. To label the cells with BSH-11R, the transformed NMuMG cells were separated with DMEM containing 5 mM ethylenediaminetetraacetic acid (EDTA) and washed with PBS, and then incubated in DMEM with 20 µM of BSH-11R at room temperature for 10 min. Then, the cells (1 × 10^8^/mL) were incubated with EasySep RapidSphere magnetic beads conjugated with anti-BSH antibody at room temperature for 30 min. The beads were washed with PBS three times with the EasySep magnet. The positively separated cells and unbound cells were harvested as “Positive” and “Negative” samples, respectively, and they were analyzed by Western blotting or a sphere formation assay.

### 2.5. Immunofluorescent Analysis and Proximity Ligation Assay

The immunostaining of cells was performed as previously described [22] with brief modifications. The cells were fixed with 4% paraformaldehyde (PFA) for 15 min, permeated with 0.05% Triton X-100 for 10 min, and blocked with 5% goat serum. Then, the samples were incubated with primary antibodies for 16 h at 4 °C and secondary antibodies for 1 h at room temperature. Primary antibodies against CD44 were purchased from Abcam (Cambridge, UK) (ab157107). Antibodies against the BSH molecule (clone#: 36-2H or 38-5C) were developed by ITM Co., Ltd. (Nagano, Japan). Secondary antibodies against murine or rabbit immunoglobulin G (IgG), labeled with Alexa fluorophores, were obtained from Thermo Fisher Scientific, Inc. (Waltham, MA, USA). After immunostaining, the samples were mounted with ProLong Diamond Antifade Mountant with/without 4′,6-diamidino-2-phenylindole (DAPI) (Thermo Fisher Scientific, Inc., Waltham, MA, USA).

A proximity ligation assay (PLA) was performed with Duolink PLA technology (Sigma-Aldrich Co. LLC, St. Louis, MO, USA). Briefly, after fixation with 4% PFA, the cells were incubated with Duolink Blocking Solution for 1 h at 37 °C and mouse anti-BSH antibody and rabbit anti-CD44 antibody for 16 h at 4 °C. Then, the samples were washed with 1x PBS three times and incubated with PLUS and MINUS PLA probes for 1 h at 37 °C. To detect the Duolink signals, the Duolink ligation and amplification kit was used.

Immunofluorescent and Duolink images were obtained using a confocal laser scanning microscope LSM780 (Carl Zeiss AG, Oberkochen, Germany).

### 2.6. Western Blotting

Western blotting was performed as previously described [23]. The cells were lysed in cell lysis buffer (20 mM Tris-HCl (pH =7.5), 150 mM NaCl, 1 mM EDTA, 1 mM Na_2_EGTA (ethylene glycol-bis(β-aminoethyl ether)-N,N,N′,N′-tetraacetic acid), 0.5% Triton X-100) supplemented with cOmplete Protease Inhibitor Cocktail (Sigma-Aldrich Co. LLC, St. Louis, MO, USA), and PhosSTOP phosphatase inhibitor cocktail (Sigma-Aldrich Co. LLC, St. Louis, MO, USA). After sonication and centrifugation (15,000 rpm, 4 °C, 10 min), the protein concentration of the supernatants was measured using a bicinchoninic acid (BCA) protein assay (Thermo Fisher Scientific, Inc., Waltham, MA, USA). The supernatants were added to 1/3 volume of 4x SDS sample buffer (240 mM Tris-HCl (pH=6.8), 8% sodium dodecyl sulfate (SDS), 40% glycerol, 0.1% bromophenol blue, 20% 2-mercaptoethanol) and boiled at 95 °C for 5 min. Then, the samples were applied to SDS-polyacrylamide gel electrophoresis (PAGE) and transferred to polyvinylidene fluoride (PVDF) membrane (Immobilon-P, 0.45 µm) (MilliporeSigma, Burlington, MA, USA). The membranes were blocked with 0.5% skim milk (nacalai tesque, Kyoto, Japan) in tris-buffered saline with tween20 (TBST, 137 mM NaCl, 2.68 mM KCl, 25 mM Tris (pH=7.4), 0.1% Tween20) and incubated with primary antibodies for 16 h at 4 °C and secondary antibodies for 1 h at room temperature. The signals were developed with Clarity Western enhanced chemiluminescence (ECL) Substrate (Bio-Rad Laboratories, Inc., Hercules, CA, USA) and detected with a ChemiDoc imaging system (Bio-Rad Laboratories, Inc., Hercules, CA, USA).

Primary antibodies against CD44 (#5640), eIF4E (#2067), eIF4A (#2013, 2490), eIF4G (#2469), poly(A)-binding protein 1 (PABP1) (#4992), eIF6 (#3833), and eRF3 (#14980) were obtained from Cell Signaling Technology Inc. (Danvers, MA, USA). Primary antibodies against CD44 (#ab157107) were obtained from Abcam (Cambridge, UK). Primary antibodies against β-actin (#A5316), glyceraldehyde 3-phosphate dehydrogenase (GAPDH) (#MAB374), and yes-associated protein (YAP)/transcriptional coactivator with PDZ-binding motif (TAZ) (#sc-101199) were purchased from Sigma-Aldrich Co. LLC (St. Louis, MO, USA), MilliporeSigma (Burlington, MA, USA), and Santa Cruz Biotechnology (Santa Cruz, CA, USA), respectively. Secondary antibodies conjugated with horseradish peroxidase were obtained from Sigma-Aldrich Co. LLC (St. Louis, MO, USA).

### 2.7. Immunoprecipitation

Immunoprecipitation was performed as previously described [18] with modification. Briefly, the cells were lysed in cell lysis buffer supplemented with cOmplete Protease Inhibitor Cocktail and PhosSTOP phosphatase inhibitor cocktail. The lysate was passed through a 23G needle syringe 10 times and centrifuged at 15,000 rpm, at 4 °C, for 10 min. After the measurement of the protein concentration, the supernatant was pre-cleaned with protein G sepharose (GE Healthcare Life Sciences, Boston, MA, USA) and added with BSH compounds (BSH or BSH-polyR, the final concentration was 20 µM each). The mixture was incubated at 4 °C for 1 h, and we further added primary antibody against BSH (the final concentration was 10 µg/mL) and incubated at 4 °C for another hour. Then, the protein G sepharose was added to the mixture, and pull-down procedures were performed according to the manufacturer’s recommendation. The precipitates were boiled in 1× SDS sample buffer at 95 °C for 5 min. After centrifugation at 15,000 rpm for 10 min, the supernatants were applied to Western blotting.

To measure the ^10^B concentration in the immunoprecipitates with anti-CD44 antibody from the mixture of cell lysates and BSH compounds, the immunoprecipitates were obtained as written above with anti-CD44 antibody (1:50, as recommended by Cell Signaling Technology Inc. (Danvers, MA, USA)), instead of anti-BSH antibody. The precipitates were boiled in HNO_3_ at 95 °C for 5 min and centrifuged at 15,000 rpm for 10 min. Then, the supernatants were measured using inductively coupled plasma-atomic emission spectrometry (ICP-AES).

### 2.8. X-ray Irradiation and Sphere Formation Assay

For X-ray irradiation, the cells were trypsinized and resuspended in N-2-hydroxyethylpiperazine-N-ethanesulfonic acid (HEPES)-buffered DMEM/F-12 (FUJIFILM Wako Pure Chemical Corporation, Osaka, Japan) supplemented with BIT 9500 and antibiotics, and irradiated at 125 keV, 15 mA to give a dose rate of 0.85 Gy/min using a Hitachi X-ray source (MBR-1520R-3) (Hitachi Ltd., Tohyo, Japan). After irradiation, the cells were briefly centrifuged, resuspended in normal DMEM/F-12 medium, and seeded on ultra-low attachment 24-well plates (#3473) (Corning Inc., Corning, NY, USA) for a sphere formation assay (1000 cells/1.5 mL/well). The numbers of spheres (diameter >100 µm) were counted 1 week after seeding.

### 2.9. Neutron Irradiation

In vitro testing was performed by using the Nagoya University Accelerator-Driven Neutron Source (NUANS)*, in which neutrons are generated using a Dynamitron accelerator and a sealed Li target [24,25]. The Dynamitron is a DC accelerator with a maximum accelerating voltage of 2.8 MV and a maximum proton current of 15 mA. This system can generate epi-thermal neutrons of 10^9^ n/cm^2^/s at the extraction port satisfying the neutron specifications for BNCT indicated in The International Atomic Energy Agency (IAEA) TECDOC-1223**. In the neutron irradiation experiment, a water phantom (20cm × 20cm × 20cm) was set in front of the extraction port to produce thermal neutrons for the neutron irradiation to cells in a micro tube. The thermal neutron flux at the irradiation point was measured by the gold-foil activation method and evaluated to be 6.5x 10^7^ n/cm^2^/s in the experiment. When the concentration of boron was 50 ppm in the cell, the boron dose on the cell was estimated at approximately 3.3 Gy-eq/h. We also confirmed that a gamma ray was less than 0.5 Gy/h in the irradiation field.

### 2.10. Gene Expression Analyses

The Cancer Genome Atlas (TCGA) dataset analyses were done in the UCSC Xena data browser (https://xenabrowser.net). The expression levels of *SLC7A5*, *CD44*, *EIF4E*, *EIF4A1*, *EIF4G1*, and *GSPT1* (which encode LAT1, CD44, eIF4E, eIF4A, eIF4G, and eRF3 proteins, respectively) were constructed as a heat map. We obtained the *CD44* and *SLC7A5* expression data of glioma tissues and normal brains from the REMBRANDT glioma dataset via betastasis (http://www.betastasis.com). The expression levels are shown in box-plot graphs.

## 3. Results

### 3.1. Cellular Uptake of BSH-polyR in Various Types of Cancers

Although early clinical studies in the 1960–1970s were not fully convincing, we believe that BSH has promising potential as a ^10^B agent for BNCT, due to its high boron content and positive data in recent clinical studies [9]. To improve the poor membrane permeability of BSH, we previously developed BSH-polyR (Figure 1A). Immunofluorescent analysis showed that BSH-11R, as well as BSH-3R, successfully passed through the plasma membrane of U251 glioma cells at 20 µM, while BSH could not (Figure 1B). As we sought to expand the possibility of BSH-polyR usage not only in glioma but also in other kinds of cancer, including breast cancer and pancreatic cancer, we asked whether BSH-polyR could penetrate the membrane of these types of cancer cell lines. We treated glioma cells (U87MG and U251MG), breast cancer cells (MCF7 and MDA-MB-231), and pancreatic cancer cells (CFPAC1 and PANC1) with BSH-11R at 10 µM for 24 h. We noticed that the efficiency of BSH-11R penetration was different among cell lines; U87MG, U251MG, MDA-MB-231, and PANC1 demonstrated BSH-11R uptake, but MCF7 and CFPAC1 did not (Figure 1C). This observation motivated us to identify the primary target molecules or the determinants of the enhanced uptake, as such information would help us select suitable patients for BSH-polyR-based BNCT in the future.

PolyR consists of serial arginine amino acids, typically between 3 and 12, and it confers cell membrane permeability on a wide variety of molecules such as proteins, peptides, and chemicals. This functional peptide originated from the discovery of the human immunodeficient virus (HIV)-derived TAT peptide (RRRQRRKKRG) [26]. The TAT-mediated cellular uptake of molecules by macropinocytosis requires a lipid raft [27,28], which consists of dense cholesterol and provides the scaffold for receptors, adhesion proteins, anchoring proteins, and certain types of glycoproteins [29,30,31]. The requirement of a lipid raft for macropinocytosis could be explained by the electrostatic interaction between the positively charged TAT peptide and negatively charged proteoglycan/glycoproteins localized on the lipid raft [27]. Past studies identified several candidates for the biding proteins of CPP. For example, Futaki and colleagues identified CXCR4 as a receptor that stimulated the macropinocytic uptake of 12R, but not 8R [17]. Yet, to our knowledge, no study has identified the universal determinants of the macropinocytosis of polyR (either short or long).

To seek the cell-surface target that determines the efficiency of BSH-polyR uptake, we focused on the difference between MCF7 and MDA-MB-231 (Figure 1C). MDA-MB-231 cells show post-EMT (epithelial–mesenchymal transition) and cancer stem cell-related traits: tumorigenic and metastatic potentials [32]. Human breast cancer stem cells are molecularly defined as a population of CD24^Low^/CD44^High^ cells [33]. CD44 is a cell-surface protein that interacts with hyaluronic acid and is involved in cell adhesion, migration, and metastasis [34,35,36]. Importantly, CD44 was reported to localize at lipid rafts on the cell surface [37,38] and exhibits several types of modifications including glycosylation [39,40]. Therefore, we examined the simple hypothesis that CD44 is a cell-surface target molecule of polyR. Consistent with this, the enhanced uptake of BSH-polyR observed in immunofluorescent analysis correlated with the Western blotting pattern of CD44 in several cell lines (Figure 1D).

### 3.2. CD44 Is Required for BSH-PolyR Cellular Uptake

**Figure 2:**
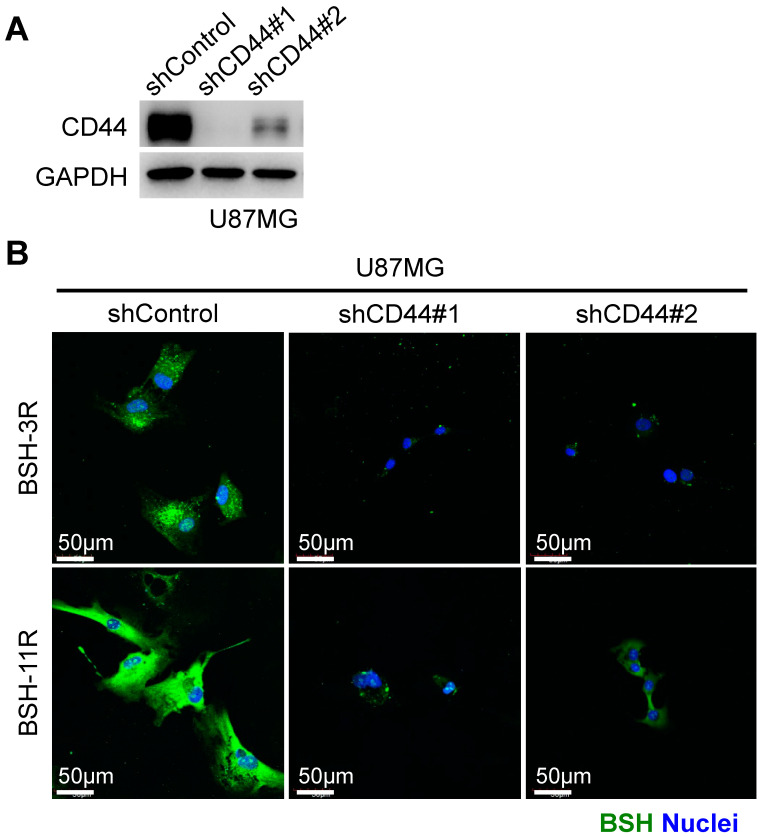
CD44 is required for cellular uptake of BSH-11R. (**A**) Lentivirus vectors expressing a short hairpin RNA (shRNA) against human CD44 worked and reduced the protein level of CD44 in U87MG cells. (**B**) CD44 depletion significantly reduced the cellular uptake of both BSH-3R and BSH-11R.

To test whether CD44 determined the enhanced cellular uptake of BSH-PolyR, we performed a loss-of-function study. We infected U87MG glioma cells with pLKO.1-puro lentiviruses carrying a negative-control shRNA or two different sequences of short hairpin RNA (shRNA) against human CD44. After 2 days of infection, the cells were selected with 1 µg/mL of puromycin for 2 days. The knockdown efficacy was validated with a Western blotting analysis (Figure 2A). Then, the selected cells were treated with 20 µM of BSH-3R or BSH-11R in DMEM with 10% FBS and antibiotics for 24 h, followed by immunofluorescent analysis using an anti-BSH antibody. BSH-3R appeared less efficient for the intracellular delivery of BSH than BSH-11R, but both in BSH-3R-treated and BSH-11R-treated cells, CD44 knockdown significantly attenuated the cellular uptake of BSH (Figure 2B).

### 3.3. Direct Interaction between BSH-PolyR and CD44 Cell-Surface Protein

**Figure 3:**
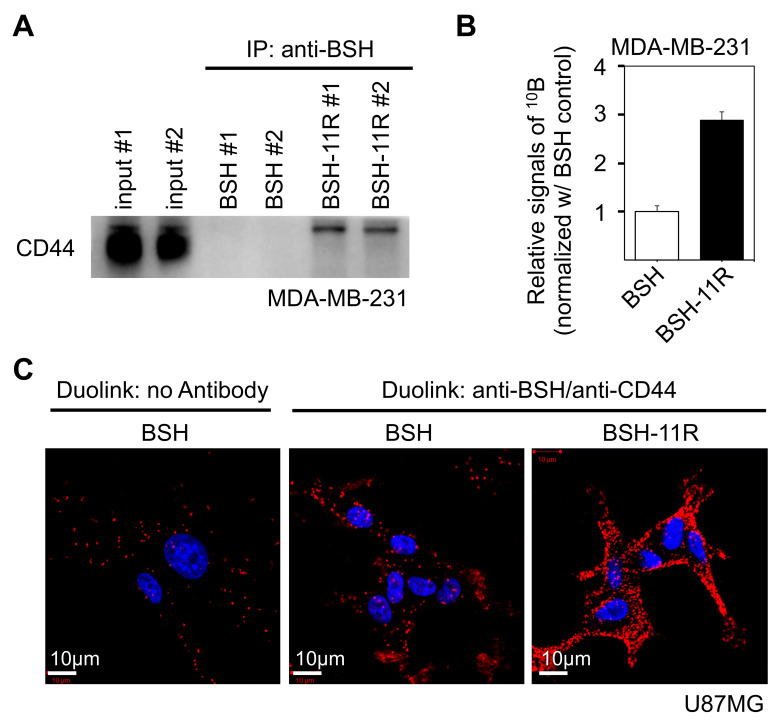
BSH-polyR directly interacted with the CD44 cell-surface molecule. (**A**) The immunoprecipitation experiment was performed with a mixture of the cell lysate of MDA-MB-231 and BSH compounds. Anti-BSH antibody successfully precipitated the CD44 molecule from BSH-11R-added samples, while it failed to precipitate CD44 from the BSH-added samples. (**B**) The ^10^B concentration of immunoprecipitates was measured using inductively coupled plasma-atomic emission spectrometry (ICP-AES). The cell lysate of MDA-MB-231 was mixed with BSH compounds and immunoprecipitated with anti-CD44 antibody. (**C**) The Duolink assay identified the in situ interaction of BSH-11R and CD44.

To ask whether BSH-polyR directly binds to the CD44 protein, we performed immunoprecipitation experiments with anti-BSH antibody. We prepared 1 mL of cell lysate of MDA-MB-231 (protein concentration was 2000 µg/mL), which abundantly expressed CD44 protein (Figure 1C), and mixed it with BSH or BSH-11R to a final concentration of 20 µM. After 1 h of incubation at 4 °C, anti-BSH antibody was added at a final concentration of 10 µg/mL and incubated for another hour at 4 °C. The BSH-bound complex was precipitated with protein G sepharose and then applied to Western blotting analysis. BSH-11R, but not BSH, successfully adsorbed the CD44 protein from the MDA-MB-231 cell lysate (Figure 3A). We noticed that the CD44 protein that specifically bound to BSH-11R was slightly shifted upper from the dominant CD44 band in the input samples, suggesting that the upper band represented a modified CD44 molecule.

Then, we asked whether the CD44 immunoprecipitate contained BSH molecules. To examine this, we performed immunoprecipitation with the anti-CD44 antibodies from MDA-MB-231 cell lysate supplemented with 20 µM of BSH or BSH-11R. The ^10^B content in the immunoprecipitates was analyzed with ICP-AES. CD44 molecules efficiently adsorbed BSH-11R, but not BSH, from MDA-MB-231 cell lysate (Figure 3B). These data indicated that BSH-11R interacted with CD44 protein in cell lysates via the polyR peptide.

To confirm that BSH-11R bound directly to CD44 molecules on cell surfaces, we performed Duolink assay (proximity ligation assay, PLA). This technique allowed us to visualize the in situ molecule–molecule interactions in cells and tissues [41], and we can detect the signals only when the two molecules are located closer than 40 nm. We treated U87MG cells with 10 µM of BSH or BSH-11R for 30 min and immediately washed the cells with PBS twice. Then, the U87MG cells were fixed and applied to a Duolink assay with anti-BSH and anti-CD44 antibodies. As shown in Figure 3C, the cells treated with BSH-11R showed much stronger signals than those treated with BSH. These data clearly showed that the 11R peptide conferred the property of CD44 interaction on BSH molecules.

### 3.4. BSH-11R Is Efficiently Delivered to Cancer Stem-Like Cells

**Figure 4:**
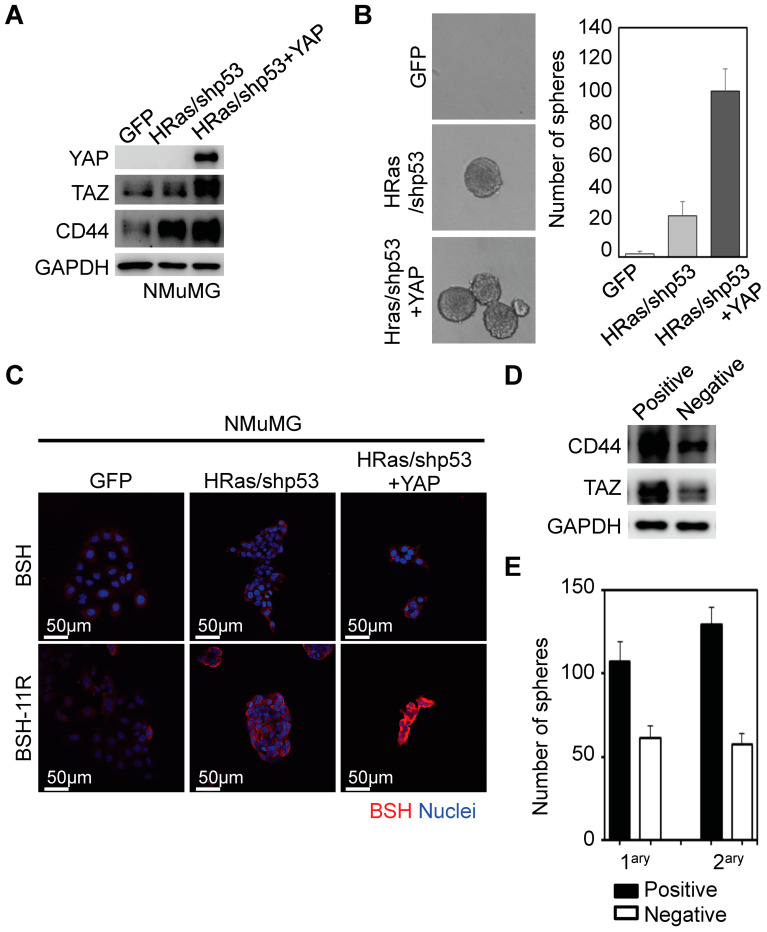
BSH-11R was efficiently delivered to cancer stem-like-cells. (**A**) Transformation with HRas and shRNA-p53 induced CD44 expression in NMuMG cells (normal murine mammary gland epithelial cells). Exogenous YAP further increased the CD44 expression level. (**B**) The transformed NMuMG cells acquired the capacity for self-renewal and anchorage-independent growth. (**C**) BSH-11R penetrated the transformed cells and efficiently accumulated in cancer stem-like cells (HRas/shp53+YAP). (**D**,**E**) BSH-11R-bound cells harbored the cancer stem cell-related traits such as abundant expression of CD44 and TAZ and the capacity for self-renewal.

As CD44 is widely accepted as one of the most reliable cancer stem cell markers in various kinds of cancer [42,43], and because BSH-polyR directly binds to the CD44 cell-surface protein as shown above, we thought that BSH-polyR could be a promising cancer stem cell-directed ^10^B agent. To test this possibility, we prepared several cell populations with cellular heterogeneity by transforming NMuMG cells (normal murine mammary gland epithelial cells) with oncogenic lentivirus and YAP1-overexpressing lentivirus (Figure 4A). The NMuMG cells were transformed upon HRas overexpression and p53 knockdown, and they acquired cancer stem cell-related properties such as the capacity for self-renewal and anchorage-independent growth (Figure 4B).

To enhance the stemness, we further overexpressed YAP1, one of the Hippo pathway transducers, because it was shown to stimulate the stemness and promote malignancy in various types of cancers [44,45]. As shown in Figure 4B, YAP1 overexpression potentiated the self-renewal capacity and CD44 protein level. We treated NMuMG-GFP (as infection control), NMuMG-HRas/shp53, and NMuMG-HRas/shp53/YAP1 cells with BSH (2 mM) or BSH-11R (10 µM) and performed immunofluorescent analyses using anti-BSH antibody. We observed that BSH treatment did not show any signals in all types of cells even at high concentrations (2 mM) (Figure 4C). On the other hand, as we expected, BSH-11R was successfully delivered into the transformed cells, especially in YAP1-overexpressing cells (Figure 4C).

Next, we demonstrated that BSH-polyR was directed to the CD44^High^ cancer cell population. To do this, we utilized NMuMG-HRas/shp53 cells, because these cells exhibited heterogeneity of the BSH staining pattern after the treatment of BSH-11R. After the separation with EDTA, the cells were treated with 20 µM of BSH-11R for 10 min at room temperature, and they were subjected to immunomagnetic separation with anti-BSH antibody and the EasySep “Do-It-Yourself” selection kit. As we expected, the positively isolated cells showed abundant expression levels of CD44 (Figure 4D). These cells were enriched with TAZ, the other Hippo transducer essential for the maintenance of stem cells in breast cancer [32]. Consistent with this, the positively isolated cells formed greater numbers of spheres compared to the negative cell population (Figure 4E). These results demonstrated that BSH-11R selectively and specifically targeted CD44^High^ cancer cells, which was usually overexpressed in cancer stem cells of various types of cancer.

### 3.5. BSH-PolyR-Based BNCT Is Effective against CD44^High^ Glioma Stem-Like Cells

**Figure 5:**
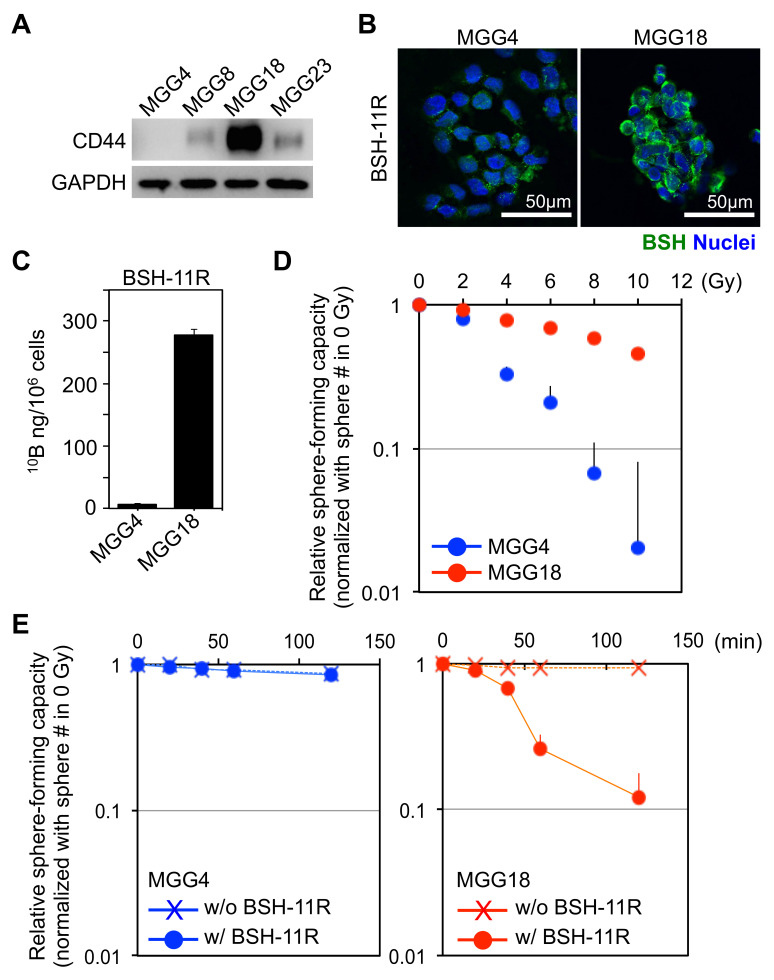
BSH-11R-based boron neutron capture therapy (BNCT) was effective against CD44^High^ glioma stem-like cells. (**A**) The CD44 profile of glioma stem-like cells. (**B**,**C**) BSH-11R was efficiently delivered into MGG18 cells. (**D**) The radiosensitivity of MGG4 and MGG18 cells. The MGG18 cells were highly resistant against X-ray irradiation. (**E**) BSH-11R-based BNCT induced remarkable cell death only in MGG18 cells.

We next sought to examine the anti-cancer effects of BSH-polyR-based BNCT against CD44^High^ cancer cells. As a model of this examination, we chose glioblastoma, because this disease was one of the most malignant tumors that required therapeutic innovation, and we believed that BNCT was one of the potential therapeutics as reported in the past study [9]. We prepared four types of glioblastoma stem-like cells, MGG4, MGG8, MGG18, and MGG23, and we first analyzed the expression levels of CD44 (Figure 5A). Among them, we selected MGG4 and MGG18 as CD44^Low^ and CD44^High^ cell models, respectively. We treated these cells with 20 µM of BSH or BSH-11R for 24 h, performed immunofluorescent analysis, and measured the ^10^B concentration. BSH-11R was taken up by MGG18 cells, but not MGG4 cells (Figure 5B), and this was confirmed by ICP-MS analysis (Figure 5C).

Before we examined the effect of BSH-polyR-based BNCT, we checked the radioresistance of these cells. To do this, we irradiated the cells using an X-ray source (MBR-1520R-3) (Hitachi Ltd., Tohyo, Japan) at serial doses of 0, 2, 4, 6, 8, and 10 Gy (dose rate = 1.0 Gy/min), in HEPES-buffered culture medium. After irradiation, the cells were seeded on ultra-low attachment 24-well plates to test the capability of anchorage-independent growth (1000 cells/1.5 mL/well). After 1 week of irradiation, we counted the numbers of spheroids (diameter >100 µm) and calculated the ratio (normalized with the number of spheroids at 0 Gy). X-ray irradiation readily killed MGG4 cells, but not MGG18, suggesting that MGG18 cells were highly radioresistant (Figure 5D).

We performed BNCT experiments with these cells and BSH compounds. We predicted that BSH-11R, but not BSH, would efficiently kill the MGG18 cells after neutron irradiation, while the MGG4 cell viability was not affected due to the lack of CD44 expression. As a neutron source, we prepared a sealed lithium target and a DC accelerator system (Dynamitron, maximum proton energy is 2.8 MeV) at Nagoya University. After treatment of the cells with BSH compounds for 24 h, we irradiated the cells with the accelerator-driven neutron at a flux of 6.5× 10^7^ n/cm^2^/s. We confirmed that the gamma ray dose was less than 0.5 Gy/h during the experiment.

After BNCT, the cells were seeded on ultra-low attachment plates and evaluated as done in the X-ray experiment. As we expected, the pretreatment of BSH-11R and neutron irradiation significantly induced cell death only in MGG18 (CD44^High^), not in MGG4 (CD44^Low^) (Figure 5E). These results clearly showed that BSH-11R could be a promising ^10^B agent for BNCT against CD44^High^ cancer cells. In MGG4 cells, which were highly radiosensitive (Figure 5D), no cytotoxic effect was observed after the neutron irradiation, suggesting that our neutron source was almost free from undesirable gamma rays.

### 3.6. Intracellular Target of BSH-11R

Next, we sought to identify the intracellular targets of polyR, as such information would help us to understand the intracellular distribution and retention capacity of polyR-conjugated molecules, which might further contribute to the therapeutic optimization of BSH-polyR-based BNCT. As shown in Figure 1, the penetrated BSH-polyR showed the cytoplasmic distribution as well as remarkable localization at the nucleoli rather than the nucleus. This observation prompted us to examine the relationship between BSH-polyR and RNA-related components, because the nucleoli were recently thought of as a site of ribosomal assembly and translation [46,47].

**Figure 6:**
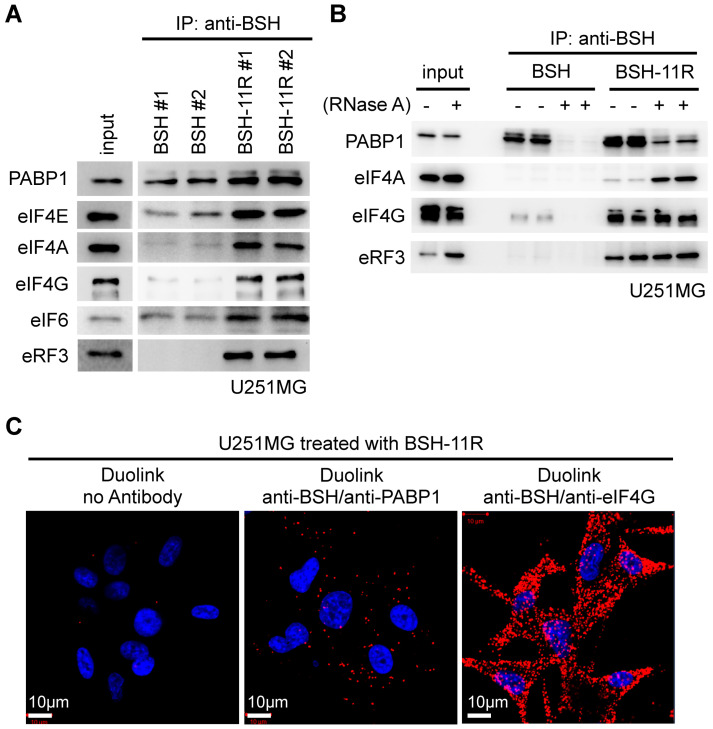
BSH-11R bound to the translation-related proteins. (**A**) BSH interacted with poly(A)-biding protein 1 (PABP1) and polyR allowed BSH to bind with the translation initiation factors and termination factor. (**B**) The interaction between PABP1 and BSH was RNA-dependent. The BSH-polyR interactions of eIF4A, eIF4G, and eRF3 appear dependent on the polyR part. (**C**) A Duolink assay confirmed the in situ interactions between BSH-11R and PABP1 or eIF4G in U251MG cells.

To examine the possibility of the interaction, we performed immunoprecipitation from the pre-cleaned lysate of U251 cells, the same as done in Figure 3A; then, we performed Western blotting using antibodies for the translation-related components. We found that BSH itself had a binding capacity with poly(A)-binding protein 1 (PABP1), which bound to the 3′ poly(A) tail of mRNA and thus controlled the translational efficiency (Figure 6A). We found that the polyR peptide precipitated translation initiation factors (eIF4E, eIF4A, eIF4G, and eIF6) and translation termination factor eRF3 (Figure 6A).

To ask whether these interactions were dependent on the RNA molecules, we incubated the pre-cleaned cell lysate with or without 10 µg/mL of RNase A for 1 h at room temperature; then, we performed immunoprecipitation and Western blotting. We revealed that BSH bound to PABP1 in the presence of RNA, while polyR-addition slightly contributed to the interaction (Figure 6B). We found that little eIF4G was pulled-down with BSH in the presence of RNA, likely via an indirect interaction through PABP1, which were known to be bound to eIF4G. EIF4G bound with BSH-polyR in the presence or absence of RNA, suggesting that the interaction was given by polyR (Figure 6B).

EIF4A did not bind to BSH in the presence or absence of RNA, but it bound to polyR, whose interaction was strengthened in the absence of RNA (Figure 6B). ERF3 did not bind to BSH, but it bound to polyR (Figure 6B). These interactions were further validated by a Duolink assay on U251 cells treated with 20 µM of BSH-11R for 24 h. As shown in Figure 6C, the combinations of anti-BSH/anti-PABP1 and anti-BSH/anti-eIF4G gave positive Duolink signals, which were not detected in the negative control samples.

### 3.7. Molecular Properties of BSH-PolyR Define the Intracellular Retention Time of BSH-PolyR

**Figure 7:**
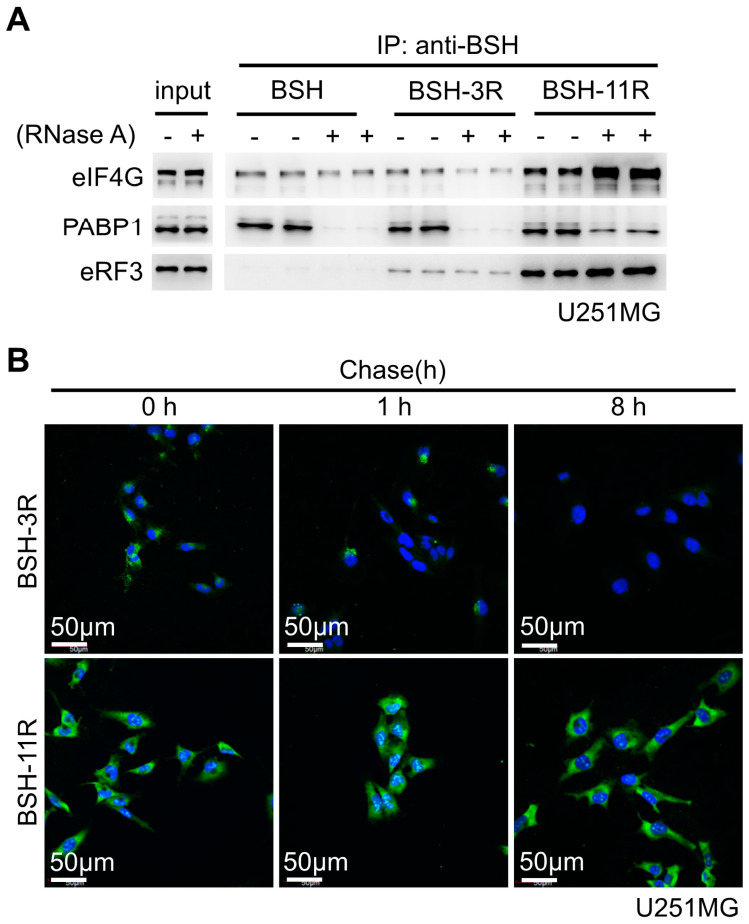
The molecular properties of BSH-polyR define the intracellular retention time of BSH-polyR. (**A**) The interactions between BSH-polyR and translation-related proteins were dependent on the length of arginine. (**B**) The polyR length affected the intracellular retention time of BSH-polyR.

We discovered that BSH was capable of binding to PABP1, and polyR to eIF4G, eIF4A, and eRF3. To ask whether the binding properties of polyR depended on the length of arginine peptides, we performed a similar immunoprecipitation experiment with 20 µM of BSH, BSH-3R, and BSH-11R. We did not observe a significant difference of eIF4G biding to BSH or BSH-3R, but we did observe an increase of the binding to BSH-11R (Figure 7A). We found that PABP1 bound to BSH in an RNA-dependent manner, and the capability of the binding to BSH compounds depended on the length of polyR (Figure 7A). We showed that eRF3 bound to arginine peptides but not BSH, and the affinity to BSH-polyR increased as the polyR length extended (Figure 7A). These data clearly showed that the polyR-peptide affected the binding capacity of BSH compounds and translation-related proteins.

Next, we examined whether the polyR length affected the intracellular retention time of BSH-polyR. To do this, we treated U251MG cells with 20 µM of BSH-3R or BSH-11R for 24 h and left them in culture medium without BSH compounds for 1 and 8 h. As shown in Figure 7B, although the starting amounts of intracellular BSH were different, the signals became invisible after 8 h in BSH-3R-treated cells, while they were still detected in BSH-11R-treated cells.

### 3.8. Bioinformatics Analyses

**Figure 8:**
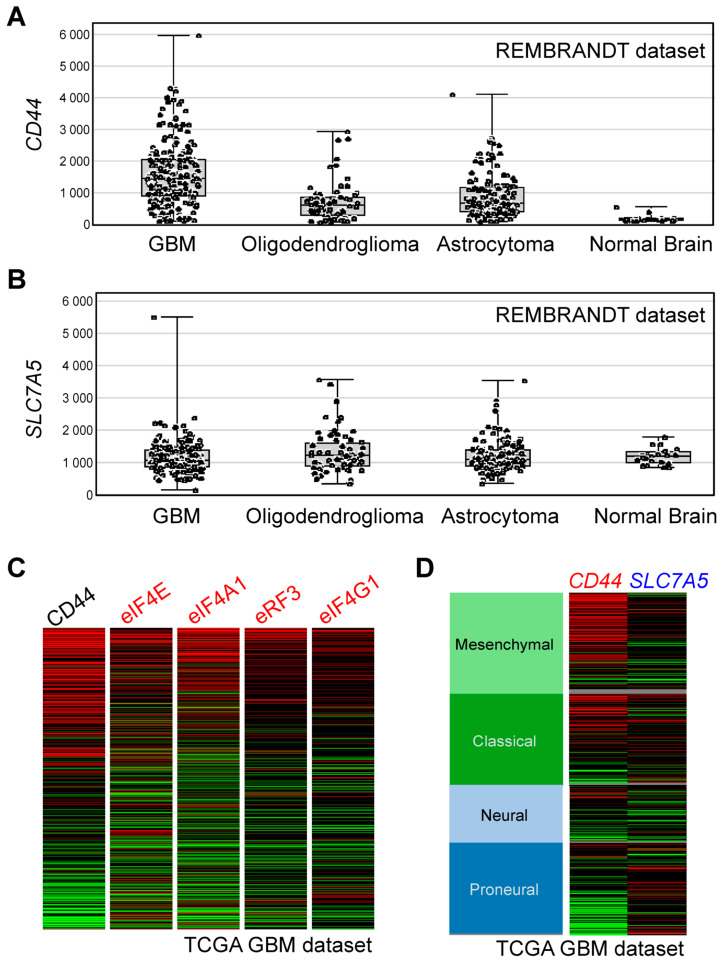
BSH-polyR may contribute to the therapeutic optimization of BSH-BNCT. (**A**) Expression levels of CD44 in brain tumor samples were higher than those in normal brains. (**B**) Profiles of the expression levels of SLC7A5 in brain tumor samples and normal brains. (**C**) Patients with high CD44 expression levels tended to harbor high expression levels of the genes encoding translation-related proteins. (**D**) Profiles of the expression levels of CD44 and SLC7A5 in the The Cancer Genome Atlas (TCGA) glioblastoma (GBM) dataset.

Thus far, we identified CD44 and the components of translational machinery as the cell-surface and intracellular targets of BSH-polyR, respectively. We propose that these properties would be helpful for selecting patients. Indeed, in the REMBRANDT brain cancer dataset, for example, the expression levels of *CD44* were increased in most glioblastoma (GBM) cases, but they were kept lower in normal brain tissues (Figure 8A). On the other hand, the expression levels of *SLC7A5* (encodes LAT1 amino acid transporter, which is responsible for the BPA uptake) in GBM, as well as in the other types of tumors, were almost the same as those in normal brain tissues (Figure 8B). Of note, in some cases of GBM, the expression levels of *SLC7A5* appeared to be lower than the normal brain. Interestingly, in the TCGA (The Cancer Genome Atlas) GBM dataset, we realized that the patients with a *CD44*^High^ signature tended to show higher expressions of the genes that encoded the proteins of the intracellular targets of BSH-polyR (eIF4E, eIF4A, eRF3, and eIF4G) (Figure 8C). This might be favorable for the intracellular retention of BSH-polyR and therapeutic optimization. Although in vivo studies using appropriate tumor models are warranted in the future, these data suggest a potential of BSH-polyR as a boron delivery agent for BNCT.

## 4. Discussion

In this study, we defined the cell-surface and intracellular targets of BSH-polyR. We identified CD44 as one of the critical determinants of BSH-polyR uptake and revealed that BSH-polyR directly bound to the CD44 molecule, using biological and biochemical techniques (Figure 2 and Figure 3). Consistent with this, BSH-polyR was readily penetrated into CD44-expressing cells, whose properties were similar to those of cancer stem-like cells (Figure 4). As a proof of principle, we performed BSH-polyR-based BNCT against the glioblastoma stem-like cells MGG4 and MGG18. BSH-polyR was successfully delivered into MGG18 (CD44^High^) but not MGG4 (CD44^Low^), and it thereby induced BNCT-dependent cell death specifically in MGG18 cells (Figure 5).

Based on the immunofluorescent finding of BSH-polyR distribution to nucleoli, we further investigated the intracellular targets of BSH-polyR and identified translational machinery-related proteins including eIF4G, eIF4A, eRF3, and PABP1 (Figure 6). Intriguingly, BSH was shown bound to PABP1 in an RNA-dependent manner, while the other proteins were shown to interact with the polyR peptide. The affinity of BSH-polyR for the proteins was dependent on the length of arginine and accordingly determined the intracellular retention time after treatment with BSH-polyR (Figure 7). The translation-related proteins as the intracellular targets of BSH or BSH-polyR suggested that BSH-based BNCT might induce selective damage to the translational machinery, which was recently proposed as a target for anti-cancer drug development [48,49]. Indeed, in the BNCT experiment in this study, we observed efficient cell death in MGG18 cells treated with BSH-polyR. This suggests that BSH-polyR-based BNCT can induce cell death in a different way from that usually is associated with X-ray irradiation.

By analyzing the expression levels of the BSH-polyR targets in a clinical dataset, we suggest that BSH-polyR might be suitable for certain types of malignant tumors. For example, in GBM, BSH-polyR appeared tumor-selective, because *CD44* expression was not typically detected in normal brain. Importantly, BSH-polyR might be useful to treat the patients with low expression levels of SLC7A5, which indicates that they are not candidates for BPA-BNCT. On the other hand, BPA might be suitable for the patients with low expression levels of CD44, which would indicate that BSH-polyR would not be a suitable boron delivery agent (Figure 8). To expand the possibility of BNCT and to accelerate the establishment of standardized medicine in the BNCT field, we need to develop novel boron agents that have different properties from BPA.

## 5. Conclusions

In the present study, we have defined the molecular properties of BSH-polyR and identified the cell-surface and intracellular targets of BSH-polyR. BSH-polyR directly binds to the CD44 cell-surface molecule for cellular uptake. Once penetrated, BSH-polyR interacts with translation-related proteins via BSH or polyR parts. As a potential boron delivery agent, we validated that BSH-polyR was effective in BNCT against CD44^High^ tumor cells. We showed that the molecular definition of the BSH-polyR-binding proteins might contribute to therapeutic optimization in the future.

## 6. Patents

A.F. is the inventor of the patent of “Cancer lesion tissue evaluation for optimizing effect of boron neutron capture therapy” (WO/2019/160129).
